# Numerical Scrutinization of Darcy-Forchheimer Relation in Convective Magnetohydrodynamic Nanofluid Flow Bounded by Nonlinear Stretching Surface in the Perspective of Heat and Mass Transfer

**DOI:** 10.3390/mi12040374

**Published:** 2021-04-01

**Authors:** Ghulam Rasool, Anum Shafiq, Marei S. Alqarni, Abderrahim Wakif, Ilyas Khan, Muhammad Shoaib Bhutta

**Affiliations:** 1 Binjiang College, Nanjing University of Information Science and Technology, Wuxi 214105, China; shoaibbhutta@hotmail.com; 2 School of Mathematics and Statistics, Nanjing University of Information Science and Technology, Nanjing 210044, China; anumshafiq@ymail.com; 3 Department of Mathematics, College of Sciences, King Khalid University, Abha 61413, Saudi Arabia; msalqarni@kku.edu.sa; 4 Mathematical Modelling and Applied Computation Research Group (MMAC), Department of Mathematics, King Abdulaziz University, P. O. Box 80203, Jeddah 21589, Saudi Arabia; 5 Laboratory of Mechanics, Faculty of Sciences Aïn Chock, Hassan II University, B.P.5366 Mâarif, Casablanca 9167, Morocco; wakif.abderrahim@gmail.com; 6 Department of Mathematics, College of Science Al-Zulfi, Majmaah University, Al-Majmaah 11952, Saudi Arabia; i.said@mu.edu.sa

**Keywords:** Darcy-Forchheimer theory, nonlinear stretching, nanofluid, magnetohydrodynamics, convective conditions

## Abstract

The aim of this research is mainly concerned with the numerical examination of Darcy-Forchheimer relation in convective magnetohydrodynamic nanofluid flow bounded by non-linear stretching sheet. A visco-elastic and strictly incompressible liquid saturates the designated porous medium under the direct influence of the Darcy-Forchheimer model and convective boundary. The magnetic effect is taken uniformly normal to the flow direction. However, the model is bounded to a tiny magnetic Reynolds number for practical applications. Boundary layer formulations are taken into consideration. The so-formulated leading problems are converted into highly nonlinear ordinary problems using effectively modified transformations. The numerical scheme is applied to solve the governing problems. The outcomes stipulate that thermal layer receives significant modification in the incremental direction for augmented values of thermal radiation parameter *R*_*d*_. Elevation in thermal Biot number *γ*_1_ apparently results a significant rise in thermal layer and associated boundary layer thickness. The solute Biot number is found to be an enhancing factor the concentration profile. Besides the three main profiles, the contour and density graphs are sketched for both the linear and non-linear cases. Furthermore, skin friction jumps for larger porosity and larger Forchheimer number. Both the heat and mass flux numbers receive a reduction for augmented values of the Forchheimer number. Heat flux enhances, while mass flux reduces, the strong effect of thermal Biot number. The considered problem could be helpful in any several industrial and engineering procedures, such as rolling, polymeric extrusion, continuously stretching done in plastic thin films, crystal growth, fiber production, and metallic extrusion, etc.

## 1. Introduction

A simple base fluid, for example, water, ethylene glycol, and oil, etc., when upgraded with the suspension of nanometric metallic strong conductive particles, is termed as nanofluid. Such a formulation sufficiently intensifies the conduction abilities of the base fluid. Numerous applications have been discovered for the so-called nanofluids in the industrial and engineering aspects as well as in bio-medicine. For example, vehicle cooling, heat exchangers, cooling and transformer cooling, electronic cooling, and many others are typical and widely used applications of nanofluids. These are also applicable in the medical treatments, especially cancer and tumor treatments, resonance imaging, and wound treatment, etc., and they are typically dependent on the conductive nature of nanofluids. Choi [1] introduced the definition of nanofluid in his experimental work where he proved that the suspension of nanoparticles in typical fluids drastically changes the thermo-physical properties of the fluid. Later on, Buongiorno [2] modeled the same concept in the perspective of convective transport of nanofluids. Adding details to the concept of nanofluids, Buongiorno emphasized the fact that the Brownian diffusion and Thermophoresis are two major slip factors in the transport of nanofluids. Afterwards, several interesting attempts have been reported by renowned researchers of fluid mechanics. For instance, Khan et al. [3] reported convection phenomena in nanofluid flow passing a linear stretching surface using the Keller–Box numerical method for the final solutions of the modeled governing problems. An interesting study of Mustafa et al. [4] disclosed an analysis on stagnation spot flow of nanofluids involving linear stretched sheet. For more details on this topic, one can see [5,6,7,8,9,10,11,12,13,14,15,16,17,18,19,20,21,22,23,24] and cross references cited therein.

Flow past a linear as well as nonlinear stretching surface relates the fluid mechanics with several important industrial and engineering setups, such as hot rolling, polymeric extrusion, continuously stretching done in plastic thin films, crystal growth, fiber production, and metallic extrusion, etc. Numerous articles are available in the literature that explain the flow caused by stretching surfaces, whether linear or nonlinear rates. The importance of linear stretching cannot be neglected but the flow caused by non-linear stretching rates have always played a significant role in the above mentioned procedures, especially in polymeric extrusion. In all such scenarios, the work of Cortell [25] is considered to be a pioneer. He considered a viscous fluid for studying heat and mass transfer developments driven by a nonlinear or linear stretching in the sheet. The prescribed wall temperature and constant wall temperature were both discussed in this study. Vajravelu [26] reported a study on exploration of heat transfer developments in a viscous fluid flow past a stretching sheet surface using power law velocity distribution with nonlinear stretching rate. Rana and Bhargava [27] reported a study on the fluid flow analysis and heat transfer aspects involving a nonlinear stretching rate in nanofluids flow over a sheet.

The type of nanofluids governed in the categorical classification of non-Newtonian fluids have gained special attention for numerous engineering and industrial applications based on their extended contributions in nuclear, chemical, metallic, polymeric, and plastic industries. Shampoos, paints, apple sauce, katchup, as well as different type of oils are typical genuine examples of non-Newtonian fluids. Having extended viscosity, the non-Newtonian fluids are best treated by involving the viscoelastic terms (second grade model), which is known as sub-categorical classification of differential non-Newtonian liquids related with the normal stress attribute. For a better understanding of this model, one can read [28,29,30,31,32,33,34] and the references cited therein.

The studies mentioned above are either concerned with a linear stretching surface with effective convective heating or nonlinear stretching sheets without the involvement of convective conditions together with the Darcy–Forchheimer model. Here, for the first time, we involve convective conditions to consider a visco-elastic and strictly incompressible nanoliquid (nanofluid) flow bounded by a nonlinear flat stretching surface. Firstly, the model shapes in mathematical form using the famous Navier stokes equations for incompressible non-Newtonian nanofluid. The leading problems are then transformed into highly nonlinear ordinary problems via suitable transformations. A numerical scheme is implemented for finding the final solutions. From now onward, fluid means means incompressible viscoelastic nanofluid. The next section will physically justify the existence of the problem and mathematical expressions with properly defined boundary conditions. The rest of the article comprises of the results and discussion, a graphical display, and concluding remarks.

## 2. Problem Formulation

**Figure 1:**
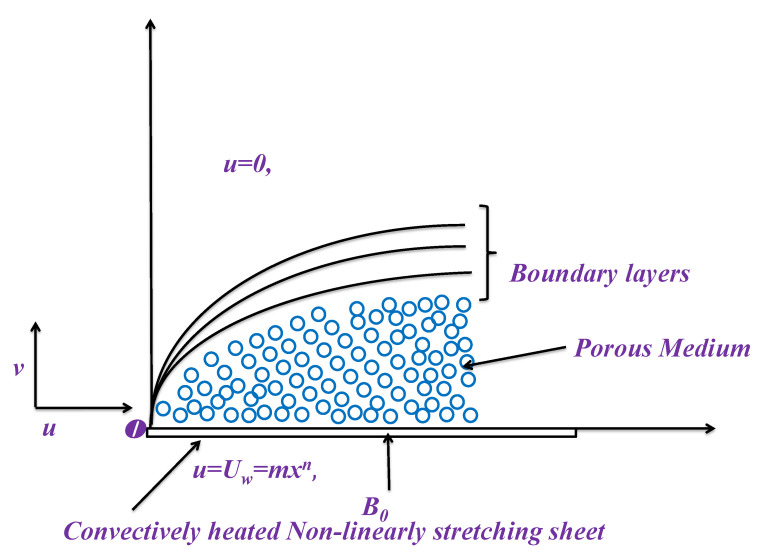
Physical model and coordinate system.

In this numerical investigation, we have invoked the convective boundary on the flow of nanofluid passing over a nonlinear flate stretching surface. These conditions are invoked to balance the temperature difference within the system. The system relies on Darcy–Forchheimer medium saturated via nanofluid over the stretching surface. Cartesian coordinates are considered to analyze the fluid flow. The flow direction is assumed along positive x–direction, whereas no-movement is allowed towards vertical. An induced magnetic effect is directly invoked to the surface normal direction via MHD; however, a tiny Reynolds number helps to dismiss the magnetic impact. The nonlinear stretching rate is taken into account via *n* as a positive integer, whereas the stretching velocity is assumed as $u=U_{w}=mx^{n}$ at the bottom line. However, the velocity diminishes away from the surface and attains zero value at free surface (u=0). The coefficient of heat and mass flux ($h_{1}$ and $h_{2}$) are involved in convective boundary conditions. Figure 1 sketches a physical display.

The modified Navier stokes problems are given below:

(1) $$\frac{\partial v}{\partial y}+\frac{\partial u}{\partial x}=0,$$

(2) $$\begin{array}{cc} v\frac{\partial u}{\partial y}+u\frac{\partial u}{\partial x}= & \nu \frac{\partial^{2}u}{\partial y^{2}}-\left[\frac{B_{0}^{2}\sigma }{\rho_{f}}+\frac{\nu }{K}\right]u-\frac{C_{b}}{K^{1/2}}u^{2}\\ \; & -k_{1}\left(u\frac{\partial^{3}u}{\partial x\partial y^{2}}+\frac{\partial u}{\partial x}\frac{\partial }{\partial y}\left(\frac{\partial u}{\partial y}\right)+v\frac{\partial }{\partial y}\left(\frac{\partial^{2}u}{\partial y^{2}}\right)-\frac{\partial u}{\partial y}\frac{\partial }{\partial x}\left(\frac{\partial u}{\partial y}\right)\right), \end{array}$$

(3) $$v\frac{\partial T}{\partial y}+u\frac{\partial T}{\partial x}=\alpha \frac{\partial }{\partial y}\left(\frac{\partial T}{\partial y}\right)+\frac{{\left(\rho c\right)}_{p}}{{\left(\rho c\right)}_{f}}\left[D_{Br}\left(\frac{\partial C}{\partial y}\frac{\partial T}{\partial y}\right)+\frac{D_{Th}}{T_{\infty }}{\left(\frac{\partial T}{\partial y}\right)}^{2}\right]-\frac{1}{{\rho c}_{f}}\frac{\partial q_{r}}{\partial y},$$

where $q_{r}$ is known as radiative heat flux. Given by Rosseland’s approximation:

(4) $$q_{r}=-\frac{\partial (T^{4})}{\partial y}\left(\frac{4\sigma^{*}}{3k^{*}}\right),$$

where $\sigma^{*}=$ Stefan–Boltzmann constant and $k^{*}=$ mean absorption, respectively. Here, in,

(5) $$4T_{\infty }^{3}T-3T_{\infty }^{4}≅T^{4},$$

confirms,

(6) $$\frac{\partial q_{r}}{\partial y}=-\frac{\partial^{2}T}{\partial y^{2}}\left(\frac{16\sigma^{*}T_{\infty }^{3}}{3k^{*}}\right).$$

Therefore, the energy equation has the following final form:

(7) $$u\frac{\partial T}{\partial x}+v\frac{\partial T}{\partial y}=\alpha \frac{\partial }{\partial y}\left(\frac{\partial T}{\partial y}\right)+\frac{{\left(\rho c\right)}_{p}}{{\left(\rho c\right)}_{f}}\left[\frac{D_{Th}}{T_{\infty }}{\left(\frac{\partial T}{\partial y}\right)}^{2}+D_{Br}\left(\frac{\partial C}{\partial y}\frac{\partial T}{\partial y}\right)\right]+\frac{\partial^{2}T}{\partial y^{2}}\left(\frac{16\sigma^{*}T_{\infty }^{3}}{3k^{*}}\right)\frac{1}{{\left(\rho c\right)}_{f}},$$

(8) $$v\frac{\partial C}{\partial y}+u\frac{\partial C}{\partial x}=D_{Br}\left(\frac{\partial }{\partial y}\frac{\partial C}{\partial y}\right)+\frac{D_{Th}}{T_{\infty }}\left(\frac{\partial }{\partial y}\frac{\partial T}{\partial y}\right),$$

Equations (1),(2),(7), and (8) are known as the governing equations with the following boundary conditions, as per the present model,

(9) $$u=U_{w}=mx^{n},\;h_{1}\left(T_{f}-T\right)=-k\frac{\partial T}{\partial y},\;v=0,\;h_{2}\left(C_{f}-C\right)=-D_{Br}\frac{\partial C}{\partial y}\;\;\mathrm{at}\;\;y=0,$$

(10) $$u=0,\;\frac{\partial u}{\partial y}=0,\;C=C_{\infty },\;T=T_{\infty }\;\;\mathrm{as}\;\;y\to \infty .$$

In the above governing equations, μ is taken as dynamic viscosity, $\nu \left(=\mu /\rho_{f}\right)$ is known as kinematic viscosity, $\rho_{f}$ is taken as density, σ is involved for electrical conductivity, $\alpha =k/{\left(\rho c\right)}_{f}$ is well known thermal diffusive force, *k* is typical thermal conductiveness, $h_{1}=h_{p}x^{\frac{n-1}{2}}$ and $h_{2}=h_{q}x^{\frac{n-1}{2}}$ are known as non-uniform heat and mass (transfer) coefficients, respectively, τ is known as the ratio given between the effective nanoparticles heating capacity versus effective fluid heating capacity, $D_{Br}$ is known for Brownian diffusion, $D_{Th}$ is thermophoretic factor, and $B_{0}$ is magnetic effect. Defining,

(11) $$\begin{array}{cc} \; & \;v=-\frac{1}{2}x^{\frac{n-1}{2}}\left[\left(\frac{n-1}{n+1}\right)\frac{\partial f}{\partial \eta }\eta +f\left(\eta \right)\right]\sqrt{2m\nu (n+1)},\;u=mx^{n}\frac{\partial f}{\partial \eta },\\ \; & \;\phi \left(\eta \right)=\frac{C-C_{\infty }}{C_{w}-C_{\infty }},\;\;\theta \left(\eta \right)=\frac{T-T_{\infty }}{T_{w}-T_{\infty }},\;\;\eta =\frac{1}{2}\sqrt{\frac{2\rho_{fl}m\left(n+1\right)}{\mu }}x^{\frac{n-1}{2}}y. \end{array}$$

Using (15) in (1), (2), (9) and (10) we have

(12) $$\begin{array}{cc} \; & f^{\prime \prime \prime }+ff^{\prime \prime }+k_{0}\left[\left(\frac{n+1}{2n}\right)f^{\prime }f^{iv}+{\left(f^{\prime \prime }\right)}^{2}-\left(\frac{n+1}{2n}\right)f^{\prime \prime \prime }f^{\prime }\right]\\ \; & -\left(\frac{2n}{1+n}\right)\left(1+F_{r}\right){\left(f^{\prime }\right)}^{2}-\lambda \left(\frac{2}{n+1}\right)f^{\prime }-M^{2}\left(\frac{2}{n+1)}\right)f^{\prime }=0, \end{array}$$

(13) $$\begin{array}{cc} \; & \left(1+4/3Rd\right)\theta^{\prime \prime }+Prf\theta^{\prime }+NbPr\theta^{\prime }\phi^{\prime }+NtPr{\left(\theta^{\prime }\right)}^{2}=0, \end{array}$$

(14) $$\begin{array}{cc} \; & \phi^{\prime \prime }+LePrf\phi^{\prime }+\frac{Nt}{Nb}\theta^{\prime \prime }=0, \end{array}$$

(15) $$\begin{array}{cc} \; & f\left(0\right)=0,\;f^{\prime }=1,\;\phi^{\prime }\left(0\right)=-\gamma_{2}\left(1-\phi (0)\right),\;\theta^{\prime }\left(0\right)=-\gamma_{1}\left(1-\theta (0)\right),\\ \; & f^{\prime }\left(\infty \right)=0,\;f^{\prime \prime }\left(\infty \right)=0,\;\phi \left(\infty \right)=0,\;\theta \left(\infty \right)=0. \end{array}$$

Here, $\gamma_{i}$ for i=1,2 are the convective parameters extracted from $h_{1}=h_{p}x^{\frac{n-1}{2}}$ and $h_{2}=h_{q}x^{\frac{n-1}{2}}$ known as non-uniform heat transfer coefficient and mass transfer coefficient, respectively. $F_{r}$ is used for inertial frame, *M* is involved for magnetic impact, λ is treated as porosity, Pr is the typical Prandtl, Le is the well known Lewis number, $k_{0}$ is taken as viscoelastic parameter, and Nb and Nt Brownian diffusion and thermophoretic factors, respectively. Mathematically,

(16) $$\begin{array}{cc} \; & \gamma_{1}=\frac{h_{p}}{k}\sqrt{\frac{2\nu }{m(n+1)}},\gamma_{2}=\frac{h_{q}}{D_{Br}}\sqrt{\frac{2\nu }{m(n+1)}},\;F_{r}=\frac{C_{b}x}{K^{1/2}},\;M^{2}=\frac{2\sigma B_{0}^{2}}{mx^{n-1}\rho_{f}},\\ \; & \lambda =\frac{2\nu }{Kmx^{n-1}},\;\mathrm{Pr}=\frac{\nu }{\alpha },\;Le=\frac{\alpha }{D_{Br}},\;k_{1}=\frac{k_{0}mx^{n-1}}{\nu },\\ \; & Nb=\frac{{\left(\rho_{f}c\right)}_{p}D_{Br}\left(C_{w}-C_{\infty }\right)}{{\left(\rho_{f}c\right)}_{f}\nu },\;Nt=\frac{{\left(\rho_{f}c\right)}_{p}D_{Th}\left(T_{w}-T_{\infty }\right)}{{\left(\rho_{f}c\right)}_{f}\nu T_{\infty }}. \end{array}$$

Furthermore, using $Re_{x}=mx^{n+1}/\nu$, the local tiny Reynolds, the non-dimensional forms of physical quantities, are given below:

$${\left(Re_{x}\right)}^{1/2}{\left(\frac{n+1}{2}\right)}^{-1/2}C_{f}=\left[\left(1-3k_{0}\right)f^{\prime \prime }\left(0\right)\right],$$

(17) $$\begin{array}{cc} {\left(Re_{x}\right)}^{-1/2}{\left(\frac{n+1}{2}\right)}^{-1/2}Nu= & -\left(1+\frac{4}{3}\right)Rd\left[\theta^{\prime }\left(0\right)\right],\\ {\left(Re_{x}\right)}^{-1/2}{\left(\frac{n+1}{2}\right)}^{-1/2}Sh= & -\left[\phi^{\prime }\left(0\right)\right], \end{array}$$

## 3. Methodology

RK45 is one of the most frequently used numerical methods for solving IVPs for its accuracy and efficiency. The built-in codes involve basic concept of converting Boundary value problems into the initial value problems and subsequent problems are solved using a parallel scheme with RK45, such as shooting technique, secant method, etc., using an appropriate set of initial guesses to approximate the solutions. Herein, the boundary value problems are converted into initial value problems together with the given boundary conditions and thereafter, careful selection of initial guess for repeated iterations of the numerical scheme are chosen to obtain the solutions.

## 4. Results and Discussion

**Figure 2:**
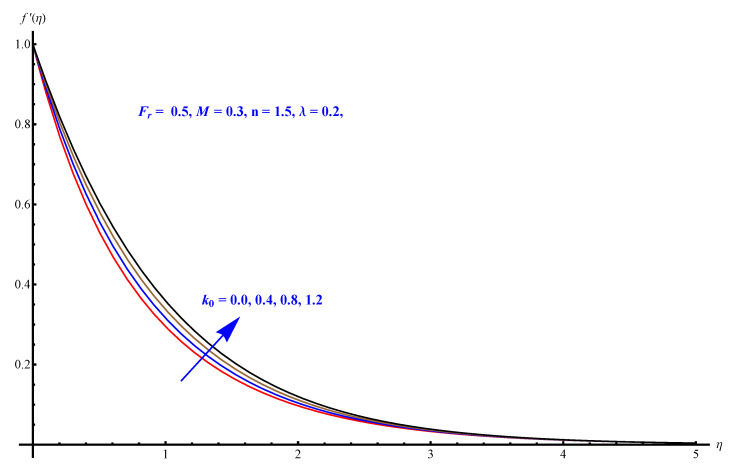
Visco-elastic parameter versus velocity field.

**Figure 3:**
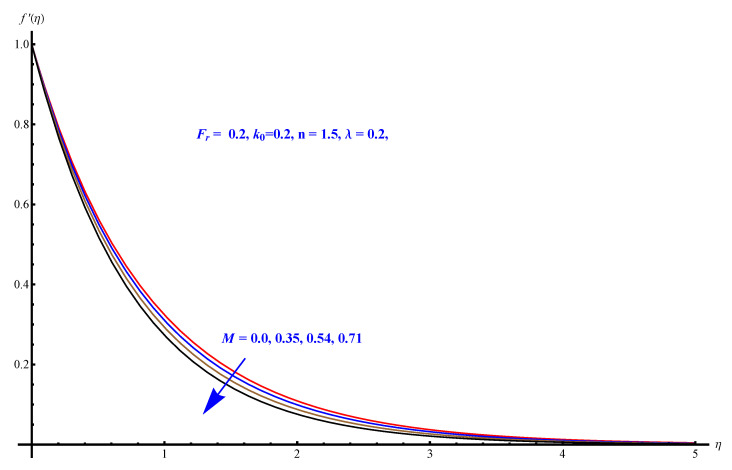
Magnetic number versus velocity field.

**Figure 4:**
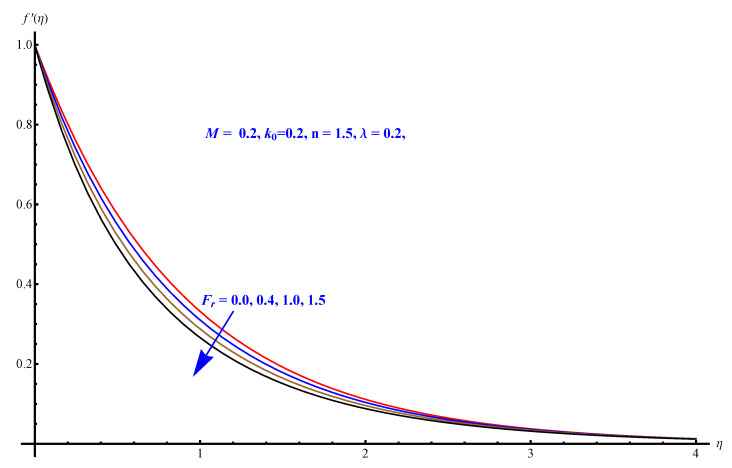
Forchheimer number versus velocity field.

**Figure 5:**
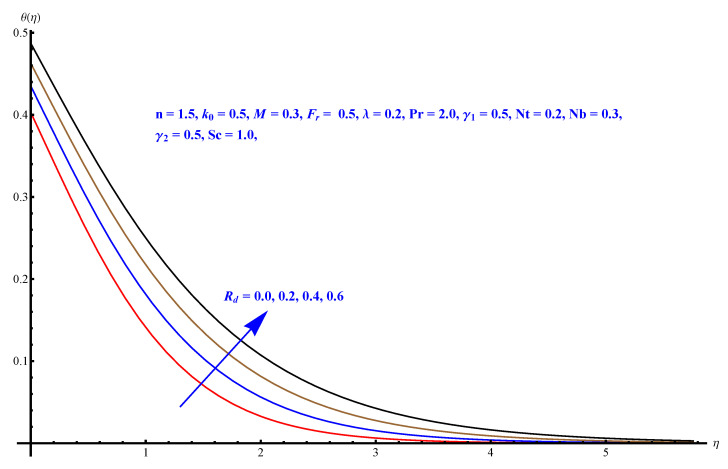
Thermal radiation parameter versus thermal profile.

**Figure 6:**
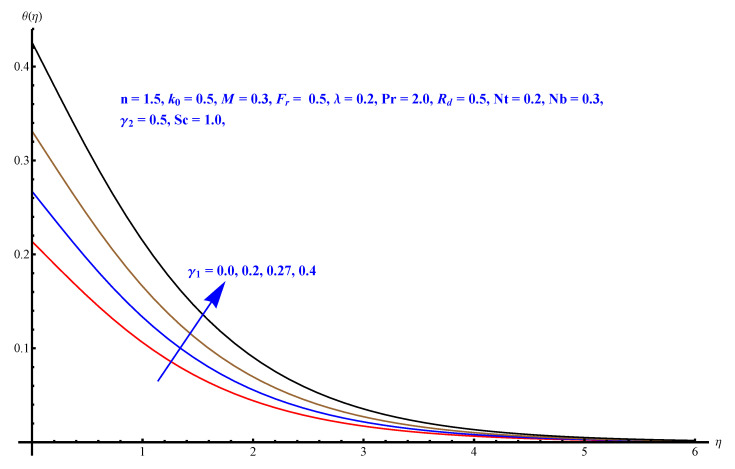
Thermal Biot number versus thermal profile.

**Figure 7:**
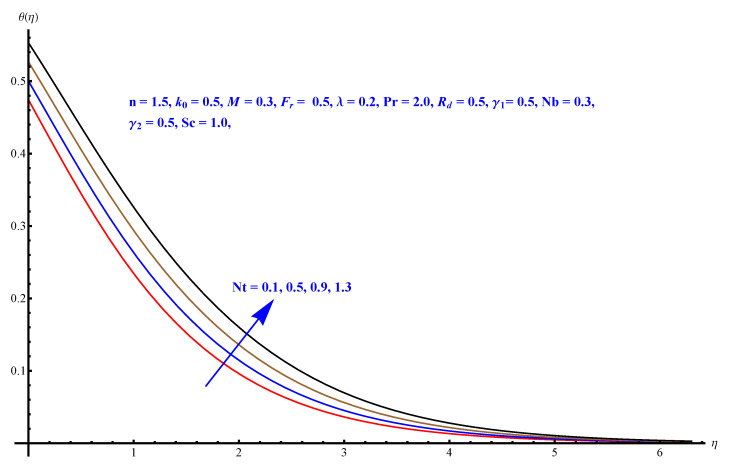
Thermophoresis versus thermal profile.

**Figure 8:**
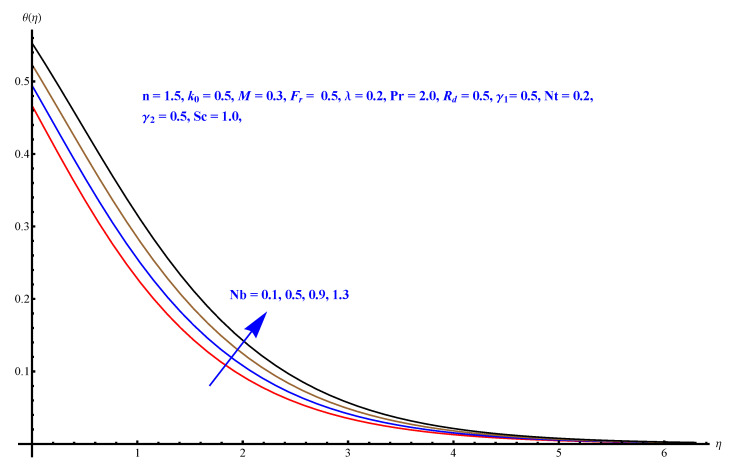
Brownian diffusion versus thermal profile.

**Figure 9:**
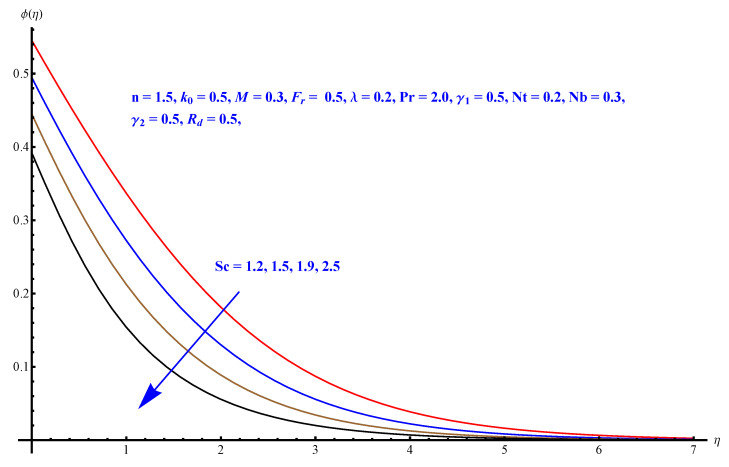
Schmidt number versus concentration profile.

**Figure 10:**
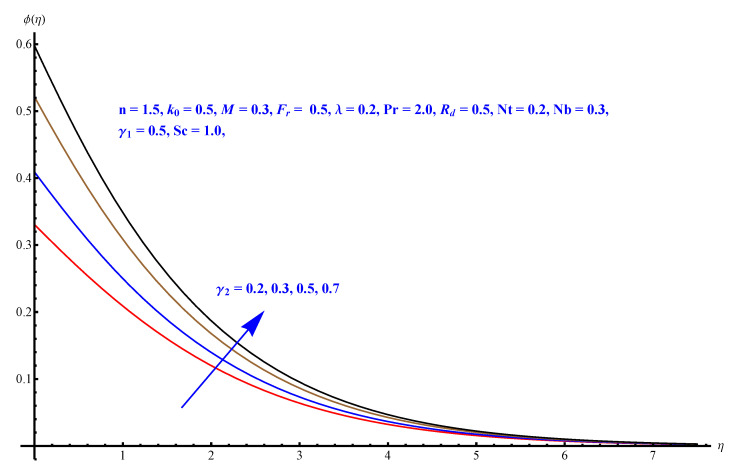
Solute Biot number versus concentration distribution.

**Figure 11:**
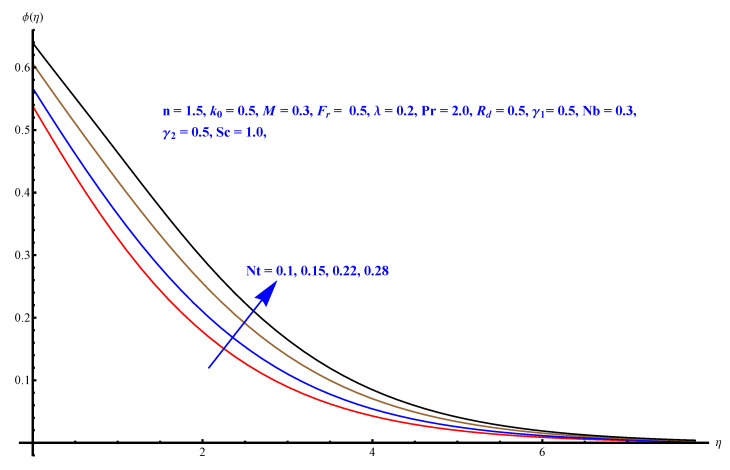
Thermophoresis versus concentration distribution.

**Figure 12:**
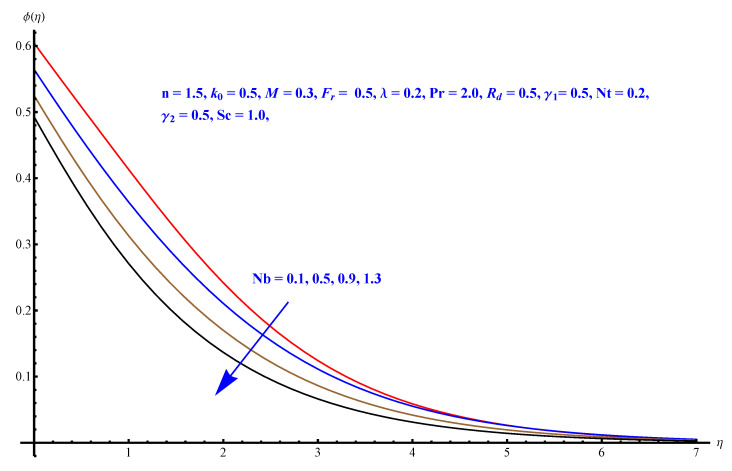
Brownian diffusion versus concentration distribution.

**Figure 13:**
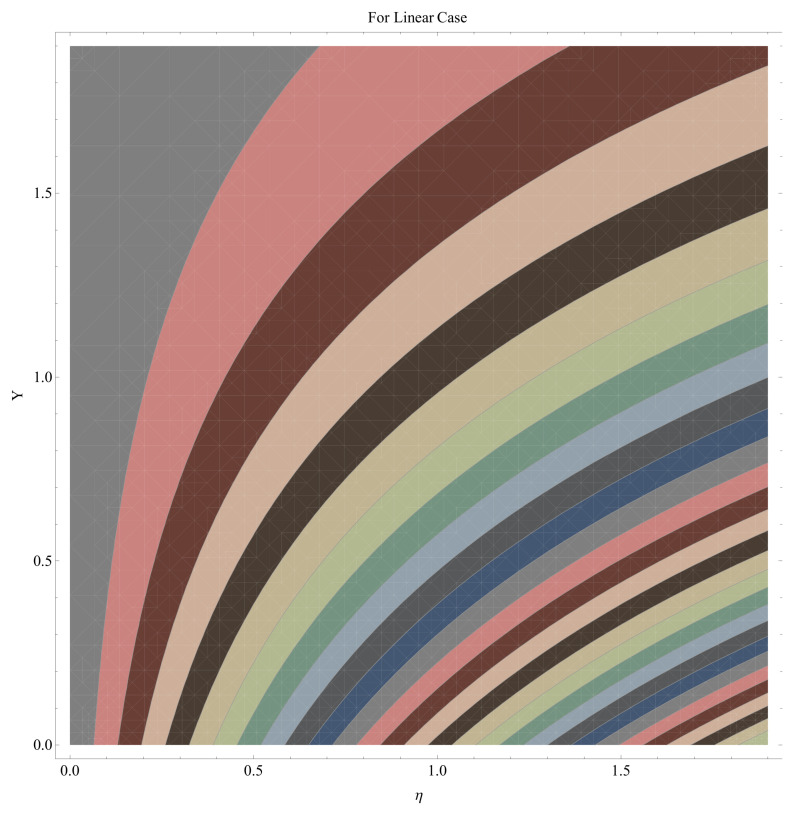
Contour graph for linear case.

**Figure 14:**
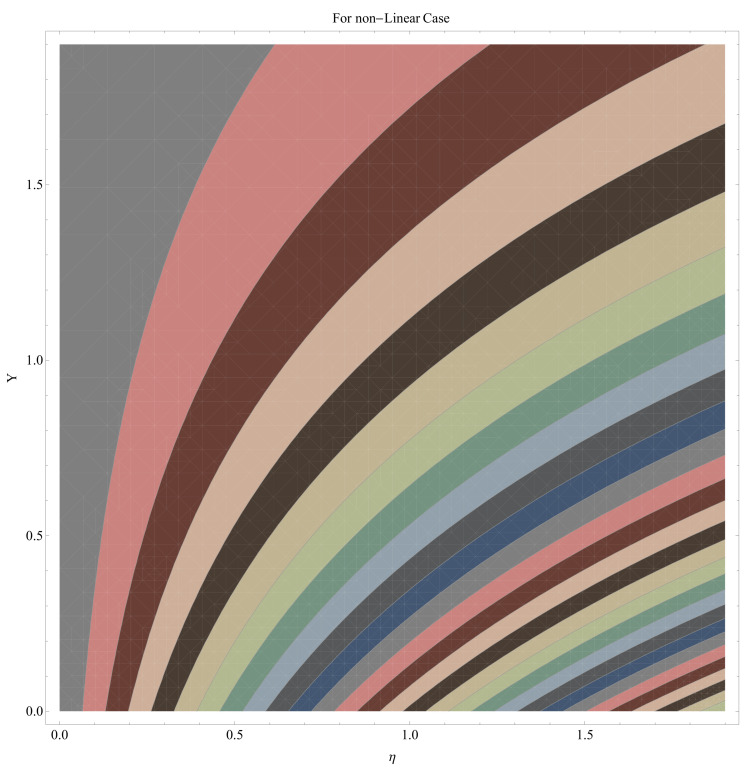
Contour graph for nonlinear case.

**Figure 15:**
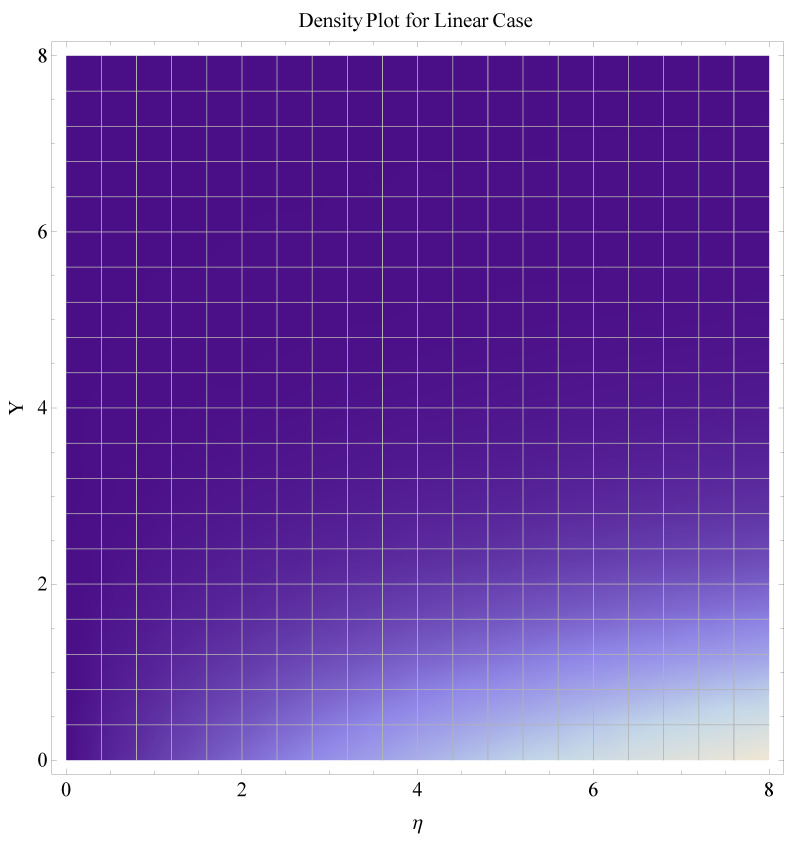
Density graph for linear case.

**Figure 16:**
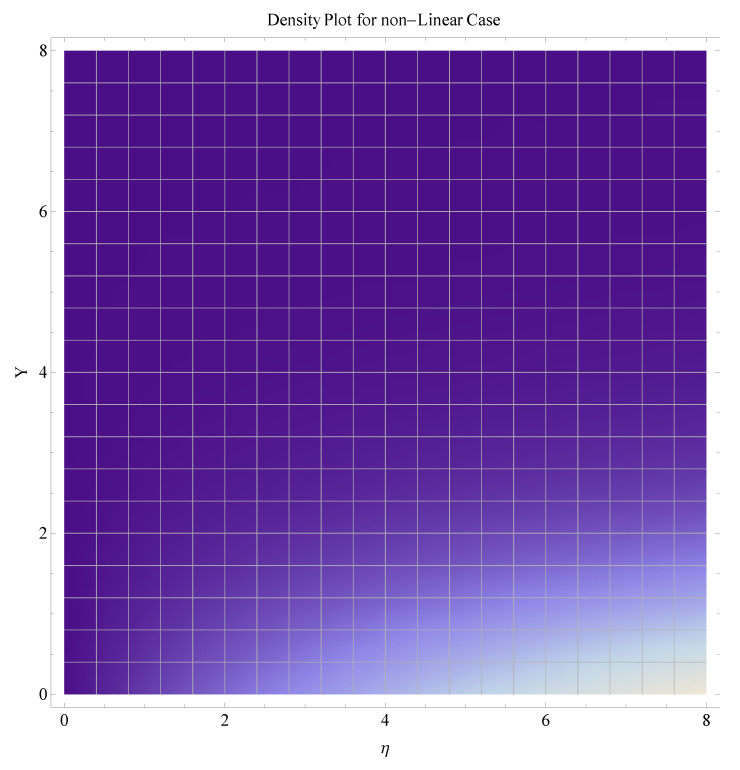
Density graph for nonlinear case.

In this section, we have described the consequences noticed in flow profiles for all relevant parameters that are involved in leading problems of present nanofluid model. The arguments are comprehended by physical justifications as to why the variation is occurred and what consequence is being witnessed. The results are obtained using the numerical method and the data are graphically plotted in increasing and decreasing curves to witness the difference. The first section of the graphs belongs to the velocity profile, the second to temperature distribution, and third is related with the concentration of nanoparticles. In particular, Figure 2 is plotted to see the variation in velocity profile against augmented trend of visco-elastic parameter $k_{0}$. The physical reasoning of the behavior noted for this parameter is connected with the combination of $k_{1}$ and kinematic viscosity μ having an inverse relation with fluid viscosity. For larger values of $k_{0}$, the fluid viscosity shows a decline, and a consequent increasing trend is witnessed in the relative profile. The magnetic parameter *M* that is given in Figure 3 with a relevant variation in fluid motion produces a reduction. Physically, the affect of magnetic field is always in anti-directional with the fluid flow because of the surface normal bumps. The certain effect of magnetic field in the direction normal to the fluid flow is, for sure, the reason behind this trend of decline in fluid velocity. The similar trend in velocity profile can be figured out for the Forchheimer number $F_{r}$ that is given in Figure 4, but this time the decline is related with a higher friction and retardation offered by porous medium. The larger the Forchheimer number, the higher the frictional force that is offered to fluid motion and the consequence is a decline in fluid velocity. The thermal layer receives significant modification in the incremental direction for augmented values of thermal radiation $R_{d}$ given in Figure 5. The influence of thermal radiation eases the way of heat flux with a more convenient convection and the corresponding profile receives significant enhancement. Physically, thermal radiation is responsible for the enhancement of the thermal state of the fluid. Elevation in thermal Biot number $\gamma_{1}$ apparently results in a significant increase of the thermal layer and associated boundary layer thickness enhances, as shown in Figure 6. The heat flux coefficient that is involved in constitution of the said parameter is a sufficient justification for this behavior of thermal profile. Thermophoresis and Brownian motion are the two important factors of nanofluid flow and boundary layer phenomenon. By definition, Brownian motion is an uncertain movement of some particles (nanoparticles in this case) in the given medium for any time t>0. The parameters are both closed influential to each other as well as on the flow profiles. Here, the thermophoresis enforces a stronger thermophoretic push to the given nanoparticles and the nanoparticles receive certain inpredictive enhanced motion and, therefore, a disturbance appears and thermal profiles increase for both of the given factors shown in Figure 7 and Figure 8, respectively. Figure 9, Figure 10, Figure 11 and Figure 12 are related with the third important flow profiles, the concentration profiles, which is solely based on the concentration of nanoparticles. In particular, Figure 9 presents the display of variation that is noticed in concentration profile with respect to the elevation in Schmidt number. Physically, the inverse relationship between Brownian diffusion and kinematic viscosity is sufficient to justify the reducing trend noticed in concentration profile for augmented values of Schmidt number. The solute Biot number is an enhancing factor the concentration profile that is given in Figure 10. The coefficient of mass flux involved in the constitutive expression justifies this variation. An enhancement is noticed for elevated values of thermophoresis given in Figure 11. A decline can be seen in concentration profile for augmented Brownian diffusion parameter given in Figure 12. For sure, the decline is based on the in-predictive and uncertain fluctuation of nanoparticles. Figure 13 and Figure 14 are the contour graphs sketched for both the linear and non-linear case, respectively, while Figure 15 and Figure 16 are the density graphs. A minor but significant difference can be seen in both the linear and non-linear case settled at n=1 & 1.5, respectively. Relevant data for skin-friction, heat, and mass flux numbers are given in Table 1 and Table 2, respectively. Skin friction jumps for larger porosity and a larger Forchheimer number, therefore, declines the easy movement of fluid.The heat flux and mass flux both receive a reduction for the augmented values of Forchheimer number. Thermal radiation is an enhancing factor for both of the flux rates. An opposite trend is noticed in both Nusselt and Sherwood numbers for thermal Biot number. Heat flux enhances, while mass flux reduces with the passage of time.

## 5. Conclusions

Here, in this research we have focused on the features of Darcy–Forchheimer relation in nanofluid flow bounded by a convectively heated non-linear stretching plane sheet. A visco-elastic and strictly incompressible nanofluid saturates the designated porous medium under the direct influence of the Darcy–Forchheimer model. The magnetic effect is taken non-uniformly normal to the flow direction. However, the model is bounded to tiny magnetic Reynolds number for practical applications. The salient findings are summarized below:

- A decreasing trend in velocity profile is noted for a stronger effect of Forchheimer number $F_{r}$.
- The thermal layer receives significant modification in the incremental direction for augmented values of thermal radiation $R_{d}$ with a more convenient convection.
- Elevated values of thermal Biot number $\gamma_{1}$ apparently result a in significant increase of the thermal layer.
- The two important factors of nanofluid flow and boundary layer phenomenon, the thermophoresis and the Brownian motion, apparently develop a rising trend in thermal profile.
- The solute Biot number is an enhancing factor for the concentration profile.
- Skin friction rises for larger porosity, while both the heat flux and mass flux receive a reduction for augmented values of Forchheimer number.
- A significant part of this study is the contour and density graphs.

## Figures and Tables

**Table 1:** Numerical data obtained for skin friction setting n=1.5.

$k_{0}$	*M*	$F_{r}$	λ	−RexCx
0.0	0.2	0.5	0.2	1.461310
0.2				0.551837
0.4				0.261381
0.1	0.0	0.5	0.2	0.984064
	0.2			0.993470
	0.4			1.021130
0.1	0.2	0.0	0.2	0.872001
		0.3		0.946589
		0.6		1.016160
0.1	0.2	0.5	0.0	0.945417
			0.2	0.993470
			0.4	1.039140

**Table 2:** Numerical data obtained for Nusselt and Sherwood numbers setting $n=1.5,k_{0}=0.1$.

*M*	$F_{r}$	λ	$R_{d}$	Pr	$N_{t}$	$N_{b}$	$S_{c}$	$\gamma_{1}$	$\gamma_{2}$	$-\theta^{\prime }\left(0\right)$	$-\phi^{\prime }\left(0\right)$
0.0	0.5	0.2	0.5	2.0	0.2	1.8	1.0	0.5	0.5	0.382563	0.284189
0.2										0.381299	0.283494
0.4										0.377592	0.281461
0.3	0.0	0.2	0.5	2.0	0.2	1.8	1.0	0.5	0.5	0.388611	0.287151
	0.3									0.383151	0.284375
	0.6									0.378095	0.281800
0.3	0.5	0.0	0.5	2.0	0.2	1.8	1.0	0.5	0.5	0.386118	0.286148
		0.3								0.376684	0.280965
		0.6								0.367978	0.276224
0.3	0.5	0.3	0.0	2.0	0.2	1.8	1.0	0.5	0.5	0.243061	0.280613
			0.3							0.326357	0.280770
			0.6							0.400512	0.281073
0.3	0.5	0.3	0.5	1.0	0.2	1.8	1.0	0.5	0.5	0.314527	0.282394
				1.5						0.353191	0.281437
				2.0						0.376684	0.280965
0.3	0.5	0.3	0.5	2.0	0.1	1.8	1.0	0.5	0.5	0.383141	0.284704
					0.3					0.370253	0.277406
					0.6					0.351170	0.267780
0.3	0.5	0.3	0.5	2.0	0.2	1.0	1.0	0.5	0.5	0.433003	0.27166
						1.6				0.390927	0.279511
						2.2				0.348077	0.283071
0.3	0.5	0.3	0.5	2.0	0.2	1.8	1.0	0.5	0.5	0.376684	0.280965
							1.5			0.377924	0.321943
							2.0			0.379042	0.348489
0.3	0.5	0.3	0.5	2.0	0.2	1.8	1.0	0.1	0.5	0.145086	0.285651
								0.3		0.298417	0.282539
								0.6		0.402833	0.280441
0.3	0.5	0.3	0.5	2.0	0.2	1.8	1.0	0.5	0.1	0.458177	0.090822
									0.3	0.408401	0.208285
									0.6	0.364879	0.307814

## Data Availability

All data are available within this manuscript.

## Nomenclature:

MHD	Magnetohydrodynamics
RK45	Runge Kutta 45 method
PDE	Partial Differential Equation
ODE	Ordinary Differential Equation
u(x,y),v(x,y)	velocity components in cartesian coordinates/m·s${}^{-1}$
$u=mx^{n}$	Horizontal velocity /m·s${}^{-1}$
*m*	constant/s${}^{-1}$
T,C	Local temperature and concentration of nanoparticles
$T_{\infty }$	Ambient temperature/K
$C_{b}$	Drag coefficient
*K*	Permeability/H · m${}^{-1}$
$B_{0}$	Magnetic field/A · m${}^{-1}$
σ	Electric conductivity/(Ohm · m)${}^{-1}$
ρ	Density/kg·m${}^{-3}$
$k_{1}$	viscoelastic coefficient
α	Thermal diffusivisity/m${}^{2}\cdot$s−1
$q_{r},Rd$	Radiation
$Nu_{x}$	Local Nusselt number
$C_{f}$	Skin-friction (wall drag force)
$Re_{x}$	Local Reynolds number
Le	Lewis number
$D_{B}$	Brownian Diffusion/m${}^{2}\cdot$ s${}^{-1}$
$D_{T}$	Thermophoretic diffusion/m${}^{2}\cdot$ s${}^{-1}$
*M*	Magnetic number
λ	viscoelastic parameter
Pr	Prandtl number
Nt	Thermophoresis parameter
Nb	Brownian diffusion parameter
τ	Ratio of heat capacity of fluid and nanoparticles
